# Decreased HAT1 expression in granulosa cells disturbs oocyte meiosis during mouse ovarian aging

**DOI:** 10.1186/s12958-023-01147-w

**Published:** 2023-10-31

**Authors:** Bichun Guo, Sainan Zhang, Shanshan Wang, Huidan Zhang, Junshun Fang, Nannan Kang, Xin Zhen, Yang Zhang, Jidong Zhou, Guijun Yan, Haixiang Sun, Lijun Ding, Chuanming Liu

**Affiliations:** 1 Center for Reproductive Medicine and Obstetrics and Gynecology, Nanjing Drum Tower Hospital, Affiliated Hospital of Medical School, Nanjing University, Nanjing, China; 2 Center for Molecular Reproductive Medicine, Nanjing University, Nanjing, 210093 China; 3 State Key Laboratory of Reproductive Medicine, Nanjing Medical University, Nanjing, China; 4 State Key Laboratory of Analytic Chemistry for Life Science, Nanjing University, Nanjing, 210093 China; 5 Clinical Center for Stem Cell Research, the Affiliated Drum Tower Hospital of Nanjing University Medical School, Nanjing, 210008 China

**Keywords:** Ovarian aging, HAT1, Meiotic defects, FoxO1, AREG

## Abstract

**Background:**

With advanced maternal age, abnormalities during oocyte meiosis increase significantly. Aneuploidy is an important reason for the reduction in the quality of aged oocytes. However, the molecular mechanism of aneuploidy in aged oocytes is far from understood. Histone acetyltransferase 1 (HAT1) has been reported to be essential for mammalian development and genome stability, and involved in multiple organ aging. Whether HAT1 is involved in ovarian aging and the detailed mechanisms remain to be elucidated.

**Methods:**

The level of HAT1 in aged mice ovaries was detected by immunohistochemical and immunoblotting. To explore the function of HAT1 in the process of mouse oocyte maturation, we used Anacardic Acid (AA) and small interfering RNAs (siRNA) to culture cumulus-oocyte complexes (COCs) from ICR female mice in vitro and gathered statistics of germinal vesicle breakdown (GVBD), the first polar body extrusion (PBE), meiotic defects, aneuploidy, 2-cell embryos formation, and blastocyst formation rate. Moreover, the human granulosa cell (GC)-like line KGN cells were used to investigate the mechanisms of HAT1 in this progress.

**Results:**

HAT1 was highly expressed in ovarian granulosa cells (GCs) from young mice and the expression of HAT1 was significantly decreased in aged GCs. AA and siRNAs mediated inhibition of HAT1 in GCs decreased the PBE rate, and increased meiotic defects and aneuploidy in oocytes. Further studies showed that HAT1 could acetylate Forkhead box transcription factor O1 (FoxO1), leading to the translocation of FoxO1 into the nucleus. Resultantly, the translocation of acetylated FoxO1 increased the expression of amphiregulin (AREG) in GCs, which plays a significant role in oocyte meiosis.

**Conclusion:**

The present study suggests that decreased expression of HAT1 in GCs is a potential reason corresponding to oocyte age-related meiotic defects and provides a potential therapeutic target for clinical intervention to reduce aneuploid oocytes.

**Supplementary Information:**

The online version contains supplementary material available at 10.1186/s12958-023-01147-w.

## Introduction

Delayed childbearing is becoming increasingly prevalent in the past decades due to the booming socioeconomic and improved educational level of women, which leads to the increased fertility demands of women over 35 years old [1]. However, the quality of oocytes significantly decreases with advanced maternal age and ovarian aging [2]. Numerous studies have reported that ovarian aging is strictly associated with abnormal oocyte meiosis, including spindle assembly malfunction, cohesin loss, or spindle deformation, which finally leads to increased oocyte aneuploidy [3, 4]. However, the mechanisms of aging-related oocyte meiotic defects have not been fully elucidated. Uncovering the mechanisms involved in regulating oocyte meiosis is crucial for aged women to supply high-quality mature oocytes for sustaining fertility.

Oocyte meiosis progression in mammals is precisely regulated. Granulosa cells (GCs) are essential for oocyte meiotic arrest and resumption [5]. Before oocyte meiotic resumption, the mural GCs produce a high level of cyclic guanosine monophosphate (cGMP) via the activation of natriuretic peptide precursor type C (NPPC)-natriuretic peptide receptor 2 (NPR2) system and transmit cGMP to the oocyte through the gap junctions, contributing to oocyte meiotic arrest [5, 6]. With the luteinizing hormone (LH) surge, binding of LH to LH receptor (LHR) triggers the release of epidermal growth factor (EGF), including amphiregulin (AREG) and epiregulin (EREG), which leads to the decrease of cGMP in GCs and oocyte meiotic resumption [7, 8]. Our previous work showed that decreased EGF levels in aged GCs are correlated with meiotic defects and aneuploidy in aged oocytes [9]. However, the potential regulatory mechanisms of the downregulation of EGF signaling in aged GCs are currently unclear.

Protein acetylation has been reported to play a crucial role in oocyte meiosis [10, 11]. However, few reports have assessed the effects of protein acetylation modifications in GCs during the process of oocyte maturation. Histone acetyltransferase 1 (HAT1) is the first identified lysine acetyltransferase, which is responsible for the acetylation of newly synthesized histone H4 on lysine 5 and 12 during chromosome assembly [12]. HAT1 is reported to be essential for mammalian development and genome stability [13]. According to the previous study, HAT1^+/−^ mice have a significantly shortened lifespan and a significant early-onset senescence phenotype [14]. In addition, the expression of HAT1 in the brain, lung, liver, and other tissues decreased significantly in the aged mice, suggesting that HAT1 may be an important factor in mammalian senescence [14]. However, the role of HAT1 in GCs or oocytes and regulatory mechanisms during ovarian aging remain unclear and need to be elucidated.

The histone acetyltransferase (HAT) activity could be inhibited pharmacologically by HAT inhibitors Anacardic acid (AA). AA is a bioactive phytochemical found in the nutshell of Anacardium occidentale, which has been reported in surprisingly broad applications ranging from antitumor, antibacterial, and so on [15, 16]. Emerging evidence suggests that AA has strong histone acetylation inhibitory effects targeted HATs, as a naturally occurring histone acetylase inhibitor [17]. Based on the above, to investigate the role of HAT1 protein acetylation modification during oocyte maturation and explore potential mechanisms in age-related oocyte meiotic defects of ovarian aging, we treated denuded oocyte (DOs) and cumulus-oocyte complexes (COCs) by AA in vitro.

In the current study, we found that HAT1 was highly expressed in GCs from young mice, whereas it was significantly downregulated in aged mice. Further, we proved that deficient HAT1 expression in GCs contributed to oocyte meiotic defects and aneuploidy via the downregulation of AREG. These data confirm that HAT1 regulates the oocyte meiosis and the downregulation of HAT1 in GCs is associated with oocyte meiotic defects and aneuploidy.

## Materials and methods

### Mice

All of the animal protocols used in the study were approved by the Experimental Animal and Welfare Ethics Committee of Nanjing Drum Tower Hospital. ICR female mice aged 3 weeks (n = 50), 6 weeks (n = 6), 10 months (n = 6), and ICR male mice aged 10 weeks (n = 10) were purchased from SPF Biotechnology Co., Ltd. (Beijing, China). All of the mice were raised under specific pathogen-free (SPF) conditions with a temperature of 20 ± 2℃, a humidity of 50–70%, a 12 h light-dark cycle, and food and water provided for free in the Animal Laboratory Center of Nanjing Drum Tower Hospital.

### Ovarian tissue immunofluorescence

Estrous cycles of mice were detected by daily examination of vaginal smears and the mice in the diestrus phase were sacrificed and ovaries were collected. The ovaries were fixed with 4% paraformaldehyde (158,127, Sigma, St. Louis, MO, USA) in PBS overnight, dehydrated in ethanol, cleared with xylene, and embedded in paraffin. The ovarian tissues were serially sectioned at 5 μm. After deparaffinization and rehydration, sections of ovarian slides were disposed in Tris-EDTA buffer (pH 9.0), and heat-induced antigen retrieval was performed. The sections were blocked with 5% goat serum after permeabilizing with 0.5% Triton X-100 (Sigma, St. Louis, MO, USA) in PBS and incubated with primary antibody against HAT1 (Rabbit / IgG, 1:200, Unconjugated, 11432-1-AP, Proteintech, China) at 4℃ overnight. We washed them by PBST, incubated them with a secondary antibody (Goat / IgG, 1:200, Alexa Fluor™ 488 conjugated, A-11,008, Sigma, St. Louis, MO, USA) in the dark for 1 h the next day, and then counterstained them with DAPI (Servicebio, Wuhan, China). Digital images were captured using a DM3000 LED microscope (Leica, Germany).

## Ovarian tissue immunohistochemistry

For the control group, ovarian were collected from female mice in the diestrus phase as described above. For gonadotropin induction ovarian, female mice were injected with 5 IU pregnant mare serum gonadotropin (PMSG) (Sansheng Pharmaceuticals, Ningbo, China), followed by 5 IU human chorionic gonadotropin (hCG) (Sansheng Pharmaceutical, Ningbo, China) 48 h later. Finally, ovarian tissues were collected after 4 h and put into 4% paraformaldehyde (158,127, Sigma, St. Louis, MO, USA) in PBS to fix. The method of preparing slices is described above. Then ovarian slides were deparaffinized, rehydrated, and disposed in Tris-EDTA buffer (pH 9.0). The sections were blocked with 5% goat serum and incubated with primary antibody against HAT1 (Rabbit / IgG, 1:200, Unconjugated, 11432-1-AP, Proteintech, China) at 4℃ overnight. Subsequently, the sections were incubated with a goat anti-rabbit secondary antibody (Goat / IgG, HRP-conjugated, PV-6001, ZSGB-BIO, Beijing, China) for 45 min at room temperature and followed by staining with a DAB peroxidase substrate kit (ZSGB-BIO, Beijing, China).

### Extraction of RNA and quantitative real-time PCR (qRT-PCR)

The ovaries were collected from 6 weeks and 10 months old ICR mice and washed with precooled saline to remove blood. 500 µL TRIzol Reagent (Thermo Fisher Scientific, Waltham, MA, USA) was added to each ovary in a 1.5 mL tube. Then they were treated with 60 W ultrasound for 3 s with 5 s off by an Ultrasonic cell grinder (Scientz-IID, Xinzhi, China). The entire operation was on the ice and lasted for 1 min. For KGN cells, after they were cultured with stimulation, the medium was discarded and the cells were washed twice with 1–2 mL precooled PBS. An appropriate amount of TRIzol Reagent (Thermo Fisher Scientific, Waltham, MA, USA) (500 µL per well in 6-well plate) was added, blown several times with a pipetting gun, transferred to RNA-specific enzyme-free EP tubes, vortexed and mixed, and left for 10 min at room temperature. The next steps were the same for both ovaries and cells. We added chloroform of 1/5 volume of TRIzol Reagent (Thermo Fisher Scientific, Waltham, MA, USA) in tubes, vortexed and mixed, and left for 10 min at room temperature. The samples were centrifuged at 15000 rpm for 10 min at 4℃, the supernatant was poured off, and 1 mL precooled 70% ethanol was added. The samples were centrifuged at 15000 rpm for 10 min at 4℃ again. The supernatant was carefully sucked off. 20 µL of DEPC water was added to measure RNA concentration. Then RNA was transcribed to cDNA by 5× All-In-One RT MasterMix (Vazyme, Jiangsu, China). Each reaction system included up to 2 µg RNA template, 2 µl AccuRT Reaction Mix (4×), and up to a total volume of 8 µl nuclease-free H_2_O. Then incubated at room temperature for 5 min and added 2 µl AccuRT Reaction Stopper (5×), 4 µl 5×All-In-One RT MasterMix, and 6 µl nuclease-free H_2_O. PCR was performed after thoroughly mixing with the amplification conditions as follows: 25℃ for 10 min, 42℃ for 15 min, and 85℃ for 5 min. After the reaction, the qRT-PCR started, containing 10 µl of SYBR-Green Mixture, 0.5 µl of Primer-F, 0.5 µl of Primer-R, 2 µl of cDNA, and 7 µl of ddH_2_O. The following primer sequences were used: *HAT1* (mouse): forward, 5’-TCTAGCTTCGCCTAGCTTCC-3’, reverse, 5’-GCAACTACTTGGCACAACCA-3’; *HAT1* (human): forward, 5’-GTGCAGTGGCATGATTGCGG-3’, reverse, 5’-CACTTTGGGAGGCCAAGGCA-3’; *18 S* (mouse): forward, 5’-ATGGCCGTTCTTAGTTGGTG-3’, reverse, 5’-CGGACATCTAAGGGCATCAC-3’; *18 S* (human): forward, 5’-CGGCTACCACATCCAAGGAA-3’, reverse, 5’-CTGGAATTACCGCGGCT-3’. All the data were normalized to the expression of *18 S* using the comparative 2^−ΔΔCt^ method.

### Western blotting

Total proteins from ovarian tissues and cultured KGN cells were extracted using Radioimmunoprecipitation assay (RIPA) lysis buffer (Beyotime, China) containing protease inhibitors and treated with ultrasound. Then we put both ovarian tissues and KGN cells at 4℃ for 30 min with rotation. After centrifuging at 12,000 rpm for 5 min, the supernatant was collected and quantified by a BCA assay kit (Beyotime, China). We performed western blotting with the same amounts of protein. It is worth mentioning that the samples in Fig. 2E and 5A were extracted from the same batch of KGN cells, which resulted in the same loading control. Then the proteins were loaded onto 10% SDS-PAGE gels, separated by it, and transferred to polyvinylidene difluoride (PVDF) membranes (Sigma, St. Louis, MO, USA). At room temperature, the blots were blocked in 5% (w/v) nonfat milk in TBST for 1 h and incubated with the following primary antibodies at 4℃ overnight: anti-HAT1 antibody (Rabbit / IgG, 1:1000, Unconjugated, 11432-1-AP, Proteintech, China), anti-Caspase 3 antibody (Rabbit / IgG, 1:1000, Unconjugated, 9662s, CST, USA), anti-Cleaved caspase 3 antibody (Rabbit / IgG, 1:1000, Unconjugated, 9661s, CST, USA), anti-Bax antibody (Rabbit / IgG, 1:1000, Unconjugated, ab32503, Sigma, St. Louis, MO, USA), anti-Bcl2 antibody (Rabbit / IgG, 1:1000, Unconjugated, 12789-1-AP, Proteintech, China), anti-β-actin antibody (Rabbit / IgG, 1:10000, Unconjugated, P30002M, Abmart, China), anti-AREG antibody (Mouse / IgG, 1:500, Unconjugated, sc-74,501, Santa Cruz Biotechnology), anti-Foxhead box transcription factor O1 (FoxO1) antibody (Mouse / IgG, 1:1000, Unconjugated, mb0093, Bioworld), anti-P-FoxO1 antibody (Rabbit / IgG, 1:1000, Unconjugated, CY6217, Abways), anti-Ac-FoxO1 antibody (Rabbit / IgG, 1:1000, Unconjugated, AF2305, Affinity), and anti-Lamin B1 antibody (Rabbit / IgG, 1:1000, Unconjugated, 12987-1-AP, Proteintech, China). The membranes were incubated by HRP-conjugated secondary antibodies (Goat / IgG, 1:10000, ZB-2301, HRP conjugated, Zsbio, China) at room temperature for 1 h after washing three times. Finally, the bands were detected using chemiluminescence (ECL) reagents. The Mean Grey Value of the target proteins was estimated by ImageJ software (NIH, Bethesda, MD, USA).

### DOs and COCs collection and in vitro maturation (IVM) culture

The ovaries collected from 3-week-old ICR female mice were cut with a blade and soaked in M2 medium (Sigma, St. Louis, MO, USA). DOs in the GV stage were obtained. COCs were isolated from the antral follicles using a disposable syringe with a 20-gauge needle. After the collection of DOs and COCs, we transferred them into MEMα maturation medium covered with liquid paraffin oil in an incubator at 37 °C under 5% CO_2_. The germinal vesicle breakdown (GVBD) and PBE rate were counted after IVM of 4 and 14 h. The MEMα maturation medium contains 10% fetal bovine serum (Gibco, Grand Island, NY, USA), 10ng/mL EGF (Sigma, St. Louis, MO, USA), and 1.5 IU/mL hCG (Sansheng Pharmaceutical, Ningbo, China). The dose of AA used to culture DOs in this study was 40 µM and the dose of AA used to culture COCs was 10 µM, 20 µM, and 40 µM respectively. Moreover, oocytes used in the following experiments including oocyte immunofluorescence, chromosome spread, in vitro fertilization (IVF), and embryo culture were from COCs of the control group and 40 µM AA-treatment group after IVM of 14 h. The details are described below. All oocyte-related procedures were performed under a stereoscopic microscope (Nikon, Shanghai, China).

### Cell culture

Human granulosa cell (GC)-like line KGN cells were cultured with DMEM/F12 (Gibco, Grand Island, NY, USA) containing 10% (v/v) fetal bovine serum (Gibco, Grand Island, NY, USA) and 1% penicillin-streptomycin (Gibco, Grand Island, NY, USA) at 37 °C in a humidified atmosphere containing 5% CO_2_.

### Small interfering RNA transfection

To knock down HAT1 expression, KGN cells were cultured in 6-well plates and COCs were cultured at MEMα maturation medium. Then they were both transfected with small interfering RNAs (siRNAs) targeting HAT1 (si-HAT1) using Lipofectamine™ 3000 (Thermo Fisher Scientific, Waltham, MA, USA) following the manufacturer’s instructions. The siRNAs sequences for HAT1 (mouse) and HAT1 (human) were 5’-CCGGGAAAGATTACTGCAA-3’ and 5’-GGAAGATTACCGGCGTGTT-3’ respectively.

### Oocyte immunofluorescence

Oocytes were fixed in PBS-buffered 4% paraformaldehyde (Sigma, St. Louis, MO, USA) for 30 min, followed by permeabilization with 0.5% Triton X-100 (Sigma, St. Louis, MO, USA) for 20 min. The oocytes were washed three times and blocked in 1% BSA for 1 h. After incubation with anti-α-tubulin (Mouse / IgG, 1:200, FITC conjugate, F2168, Sigma, St. Louis, MO, USA) at 4℃ overnight, the oocytes were washed three times again as said before. Then we incubated them with DAPI (Servicebio, Wuhan, China) for 10 min at room temperature and washed them 3 times again. Finally, after the oocytes were mounted on glass slides, a DM3000 LED microscope (Leica, Germany) was used to observe.

### Chromosome spread

Oocytes were exposed to Tyrode’s buffer (Sigma, St. Louis, MO, USA) to remove the zone pellucida. Then they were transferred to the M2 medium (Sigma, St. Louis, MO, USA) once observing the disappearance of zone pellucida. After being washed in M2 medium for 5 min, about 20 oocytes were ruptured in 20 µl spreading solution on the cover slide. They were dried completely in a ventilated place, washed three times by PBST, used DAPI (Servicebio, Wuhan, China) to dye chromosomes, and covered with coverslips. Finally, the number of spread chromosomes could be counted under the DM3000 LED microscope (Leica, Germany). The spreading solution includes 1% paraformaldehyde (Sigma, St. Louis, MO, USA), 0.15% Triton X-100 (pH = 9.2) (Sigma, St. Louis, MO, USA), and 3 mM Dithiothreitol (DTT).

### IVF and embryo culture

A 10-week-old male ICR mouse was sacrificed to obtain sperm and incubated for 1 h for capacitation in the human tubal fluid (HTF) medium (MR-070, Merck Millipore). The sperm density was observed by absorbing 1 µl semen from the droplet edge, diluting 100 times with PBS, and measuring concentration with a blood cell counting plate. Then dispersed sperm and the MII oocytes were added to 50 µl HTF (Merck Millipore). The amount of semen added was calculated by the sperm density measured before to ensure the sperm was at 1 × 10^6^/ mL. After 4 to 6 h of oocyte-sperm coincubation, zygotes were washed with the pipette. Fertilized oocytes were transferred into KSOM (MR-106-D, Merck Millipore) and cultured until the blastocyst stage. The two-cell embryos and blastocyst formation rate were calculated at 1 and 4 days after fertilization respectively.

### Single oocyte RNA sequencing and analysis

After IVM with (control groups, n = 3) or without (AA groups, n = 3) AA for 6 h, COCs were digested by hyaluronidase (H3506, Sigma, St. Louis, MO, USA) to obtain oocytes. A Discover-sc™ WTA Kit V2 (N711, Vazyme, Jiangsu, China) was used to reverse-transcribe total oocyte RNA into cDNA according to the manufacturer’s instructions. A TruePrep™ DNA Library Prep Kit V2 for Illumina (TD503, Vazyme, Jiangsu, China) was used to construct the libraries. Library sequencing and analysis were performed by Illumina HiSeq X platform (Shanghai, China). The RNA-seq data were analyzed to observe the whole clustering profile by the psych package in R. The PCA selected highly variable genes (coefficient of variation > 1) and the PCA plot was mapped using the ggplot2 package in R studio. DEGs were identified using a DESeq2 package. GO enrichment analysis was performed with the database for Annotation, Visualization, and Integrated Discovery (DAVID).

### Analysis of the mitochondrial membrane potential (MMP)

KGN cells were incubated in DMEM/F12 medium (Sigma, St. Louis, MO, USA) with JC-1 assay kit (Invitrogen, Carlsbad, CA) in the dark at 37 °C for 30 min, followed by 3 washes with PBS. The fluorescence intensities of green fluorescent J-monomers and red fluorescent J-aggregates were captured by fluorescence microscopy (Leica Germany). The fluorescence intensities of KGN cells were estimated by ImageJ software (NIH, Bethesda, MD, USA).

### JASPAR bioinformatic analysis

JASPAR (http://jaspar.genereg.net/) database was used to predict and generate a visual analysis of the transcription factor (TF) binding to AREG promoter region.

### Luciferase reporter assay

Based on the mouse AREG mRNA sequences in GenBank, the promoter of AREG was amplified and cloned into a pGL3-promoter luciferase reporter vector. KGN cells were co-transfected with AREG-promoter *or/*and pCMV-flag-FoxO1 vectors, together with luciferase plasmids. After 24 h, the cells were lysed using RIPA buffer. The Dual-Glo dual luciferase reporter assay system (Promega, Beijing, China) was utilized here to analyze and calculate the ratio of luminescence intensity.

### Statistical analysis

All analyses were performed using GraphPad Prism 9.0 statistical software (San Diego, CA, USA) and statistical comparisons were analyzed by Student’s t test (*P < 0.05, ** *P* < 0.01, *** *P* < 0.001, **** *P* < 0.0001). Data are presented as the mean ± SEM. *P* < 0.05 was considered statistically significant.

## Results

### HAT1 expression is abnormally downregulated in aged GCs

**Fig. 1:**
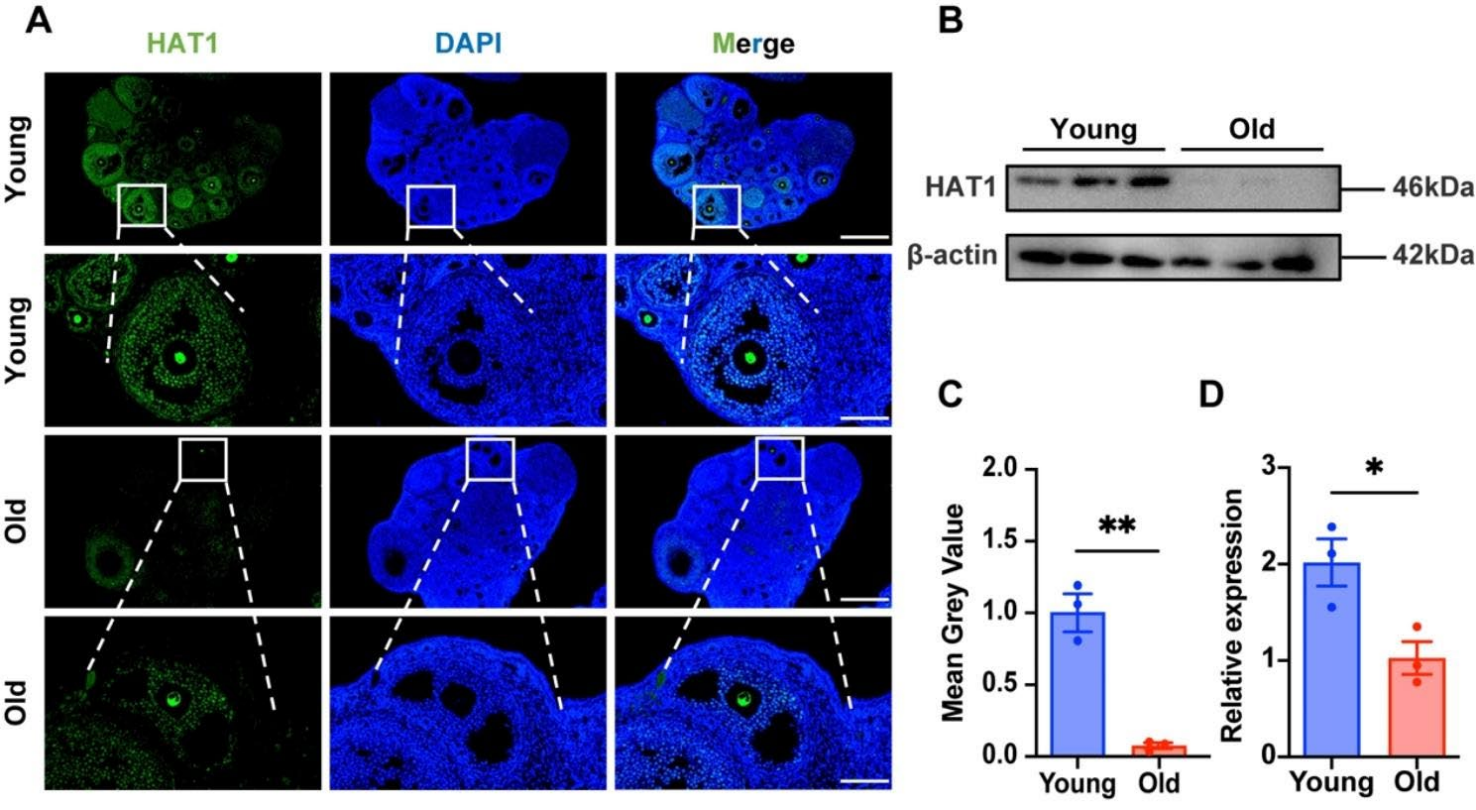
HAT1 expression is abnormally downregulated in aged GCs. **(A)** Immunofluorescence of young (6 week) and old (10 month) ovaries stained with HAT1 (green) and DAPI (blue). Scale bar: 500 μm. The amplified views of the boxed area are shown at the bottom. Scale bar: 100 μm. The experiments were repeated three times independently with similar results. **(B)-(C)** HAT1 protein expression in ovaries from young (6 week) and old (10 month) mice. The experiments were repeated three times independently with similar results. **(D)** mRNA levels of *HAT1* in ovaries from young (6 week) and old (10 month) mice. The experiments were repeated three times independently with similar results. Data are shown as the mean ± SEM, *P < 0.05, **P < 0.01, Student’s t test

To elucidate the localization and expression of HAT1 during ovarian aging in mice, we first used the ovaries of 6-week (young) and 10-month (old) mice in the diestrus phase to observe the subcellular localization and expression levels of HAT1 by immunofluorescence and immunohistochemistry. As shown in Fig. 1A, HAT1 was specifically highly expressed in oocytes and GCs. HAT1 in GCs could be upregulated by follicle-stimulation hormone (FSH) and LH in vivo, demonstrating its potential role in oocyte maturation in response to gonadotropin induction (Figure S1). Moreover, the expression of HAT1 in GCs from aged mice was markedly lower than that from young mice in protein (0.08 ± 0.02 vs. 1.00 ± 0.13, *P* = 0.0024, Fig. 1B, C) and mRNA (1.03 ± 0.17 vs. 2.02 ± 2.05, *P* = 0.03, Fig. 1D) levels. Taken together, these results suggest that HAT1 may participate in the regulation of oocyte maturation, and the downregulation of HAT1 in GCs may be involved in ovarian aging.

### HAT1 inhibition in GCs disturbs oocyte maturation

**Fig. 2:**
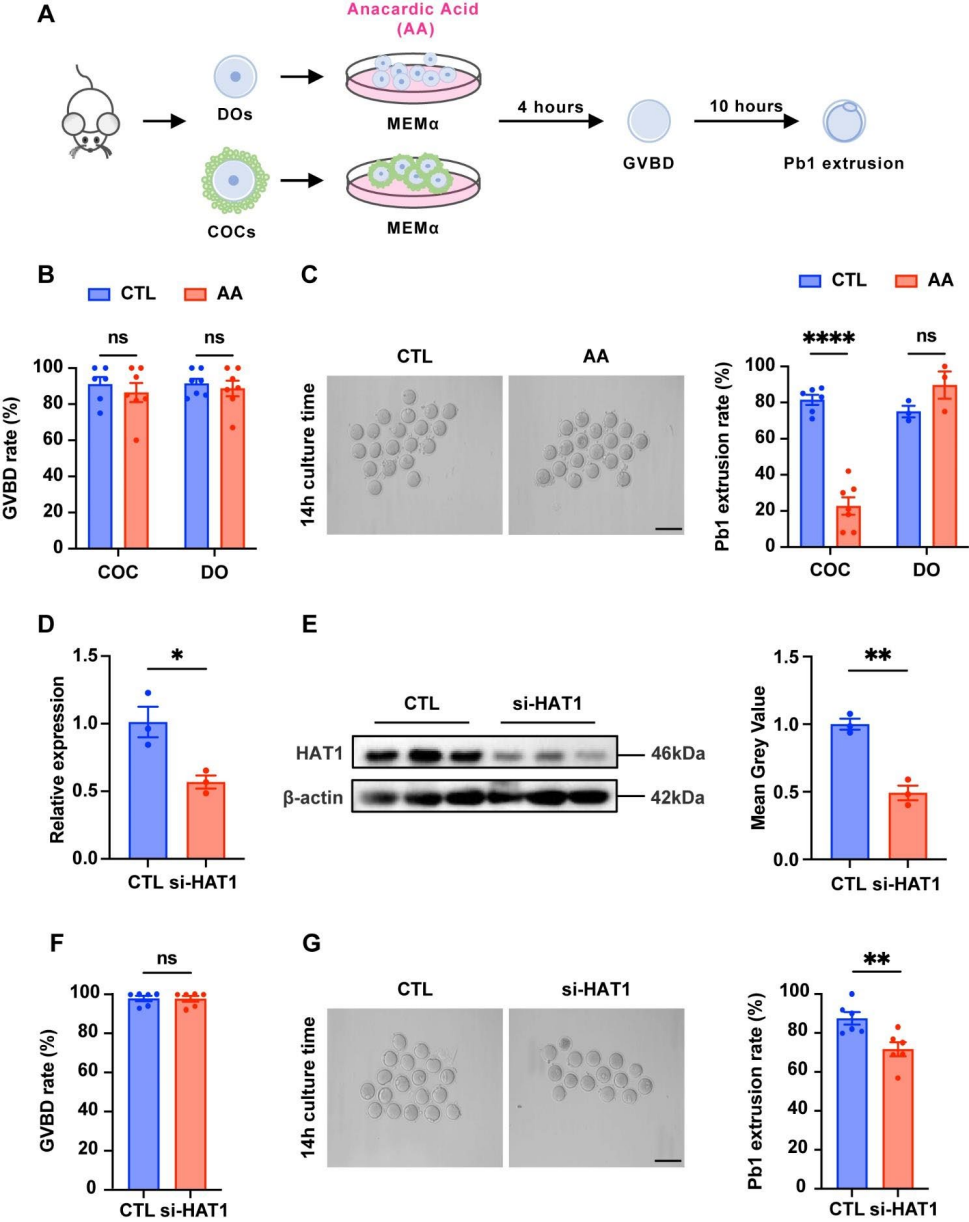
HAT1 inhibition in GCs disturbs oocyte maturation. **(A)** Schematic illustration of in vitro maturation of COCs and DOs. **(B)** GVBD rate of COCs and DOs in the control (CTL) (COCs: n = 94, DOs: n = 116) and AA-treatment (AA) (COCs: n = 101, DOs: n = 123) groups. CTL: MEMα maturation medium with 40 µM DMSO; AA: MEMα maturation medium with 40 µM AA. **(C)** Left: Representative micrographs of PBE oocytes from COCs of the CTL and AA groups. Scale bar: 200 μm. Right: Quantification of the PBE rate of COCs and DOs in the CTL (COCs: n = 98, DOs: n = 113) and AA (COCs: n = 99, DOs: n = 118) groups. **(D)** The efficiency of siRNA (HAT1) in KGN cells by qRT‒PCR. The experiments were repeated three times independently with similar results. **(E)** Western blot analysis of the expression of HAT1 in the CTL and si-HAT1 group. The samples used to examine both the efficiency of si-HAT1 knockdown (Fig. 2E) and the level of apoptosis (Fig. 5A) were from the same batch of KGN cells, which leads to the same loading control. The experiments were repeated three times independently with similar results. **(F)** GVBD rate in the CTL (n = 74) and si-HAT1 (n = 77) groups. **(G)** Left: Representative micrographs of the PBE oocytes in the CTL and si-HAT1 groups. Scale bar: 200 μm. Right: Quantification of the PBE rate in the CTL (n = 69) and si-HAT1 (n = 74) groups. Data are presented as mean ± SEM, ns, no significance, *P < 0.05, **P < 0.01, ****P < 0.0001, Student’s t test

To investigate the function of HAT1 during the progress of oocyte meiosis and maturation, DOs and COCs obtained from 3-week-old mice were treated with AA, a potent and highly selective HAT1 inhibitor [18], as displayed in Fig. 2A. Then the GVBD rate and the PBE rate of DOs and oocytes retrieved from COCs were calculated after culture for 4 and 14 h (Fig. 2A). 40 µM AA treatment did not affect the GVBD rate in both DOs (88.71% ± 4.33% vs. 91.43% ± 2.64%, *P =* 0.60) and oocytes gathered from COCs (87.17% ± 6.19% vs. 91.00% ± 4.08%, *P* = 0.52) (Fig. 2B), which implicates that AA does not disrupt GVBD. Whereas, the PBE rate of oocytes from COCs significantly reduced compared with the control group (21.17% ± 5.38% vs. 81.50% ± 2.83%, *P* < 0.0001) (Fig. 2C). Interestingly, the PBE rate of DOs had no significant difference between the two groups (89.67% ± 7.54% vs. 72.00% ± 4.51%, *P* = 0.15) (Fig. 2C), indicating that inhibition of HAT1 in GCs disturbs the oocyte meiotic maturation. When COCs were treated with AA at a dose of 10 µM, 20 µM, and 40 µM, the rate of GVBD remained constant as previously (Fig. 2B and Figure S2A). However, treatment of COCs with 10 µM, 20 µM, and 40 µM AA decreased the rate of the PBE from 71.33% ± 5.78% (*P* = 0.041) to 55.33% ± 5.78% (*P* = 0.0026), and 21.17% ± 5.38% (*P* < 0.0001) respectively compared with the control groups (89.00% ± 1.53%), indicating that AA treatment reduces the PBE in a dose-dependent manner (Fig. 2C and Figure S2B).

To further clarify the roles of HAT1 in oocyte meiosis, we used si-HAT1 to inhibit HAT1 expression (Fig. 2D, E). Consistent with the above results, the PBE rate (71.67% ± 3.64% vs. 87.50% ± 3.22%, *P* = 0.0086) declined when HAT1 expression was downregulated in GCs (Fig. 2F, G).

### HAT1 inhibition induces meiotic defects and decreases oocyte quality

**Fig. 3:**
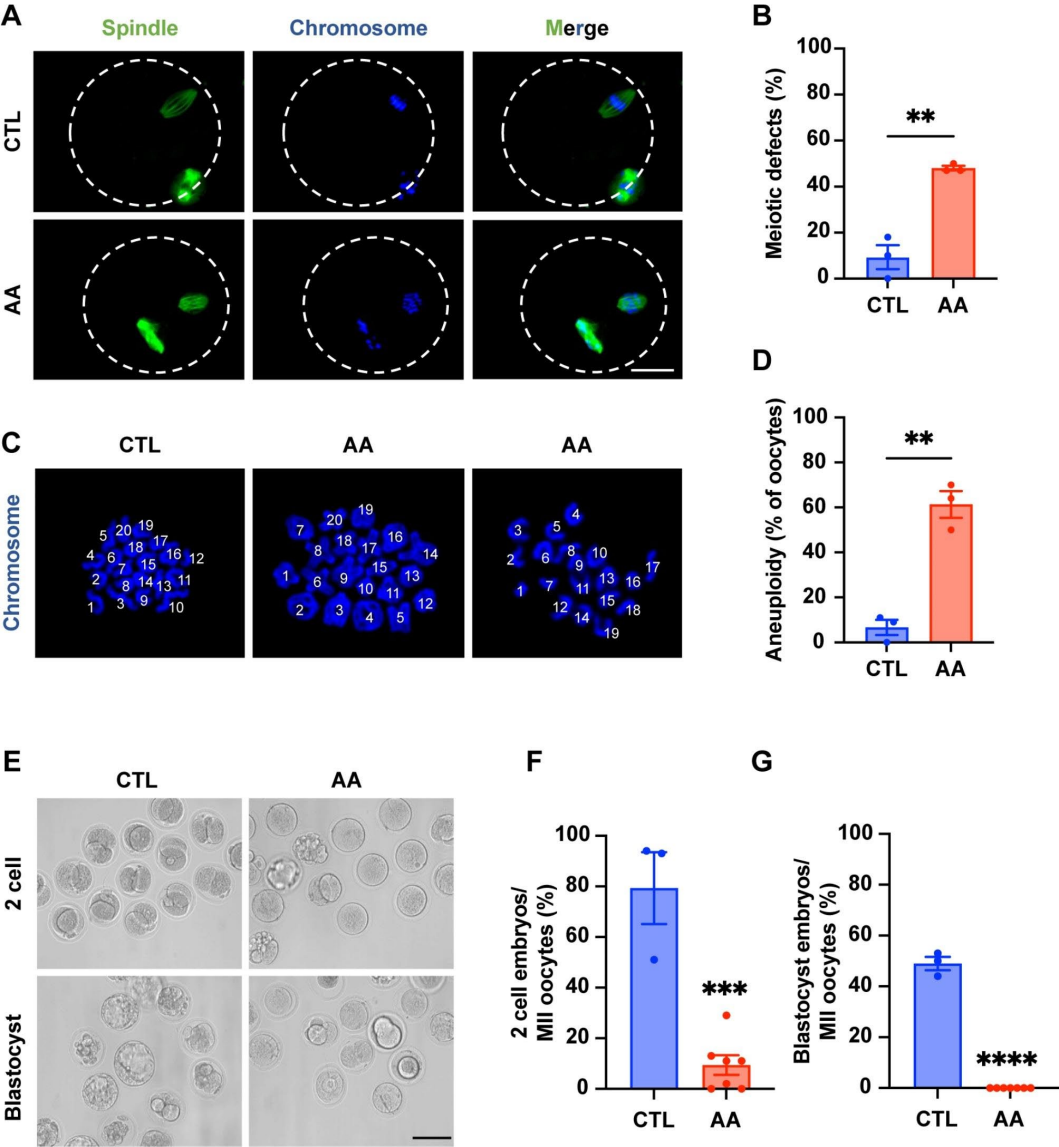
HAT1 inhibition induces meiotic defects and decreases oocyte quality. **(A)** Morphology of spindles and chromosomes in oocytes from the CTL (n = 30) and AA (n = 46) groups. Scale bar: 25 μm. **(B)** Statistical analysis of meiotic defects rate. The data are shown with three independent experiments. **(C)** Morphology of spread chromosomes in oocytes from the CTL (n = 27) and AA (n = 31) groups. Chromosomes were stained with DAPI (blue). **(D)** Statistical analysis of aneuploidy. The data are shown with three independent experiments. **(E)** Representative images of 2 cell embryo and blastocyst embryo in the CTL (n = 83 MII oocytes) and AA (n = 88 MII oocytes) groups. Scale bar: 100 μm. **(F)-(G)** Percentage of 2 cell embryos and blastocysts. The data are shown with at least three independent experiments. Data are presented as mean ± SEM, **P < 0.01, ***P < 0.001, ****P < 0.0001, Student’s t test

We then evaluated the spindle and chromosome structure in oocytes from 40 µM AA-treated COCs. We found that the control oocytes retrieved from COCs formed a standard bipolar spindle apparatus with well-aligned chromosomes at the equatorial plate. By contrast, irregularly assembled spindles and misaligned chromosome rates were markedly higher in MII oocytes gathered from AA-treated COCs than in the controls (48.00% ± 1.00% vs. 9.33% ± 5.21%, *P* = 0.0019) (Fig. 3A, B). Moreover, compared to control MII oocytes, an approximately tenfold increase in the aneuploidy rate was detected in MII oocytes from COCs treated with AA (61.33% ± 5.93% vs. 6.67% ± 3.38%, *P* = 0.0013) (Fig. 3C, D). The results of si-HAT1 also demonstrated the existence of abnormal spindles and chromosomes with the depletion of HAT1 in GCs (Figure S2C, D).

We further tested the fertilization capacity of oocytes from control and AA-treated COC groups. The results showed that most oocytes derived from AA-treated COCs could not develop into 2-cell embryos (9.43% ± 3.88% vs. 79.33% ± 14.17%, *P* = 0.0002) and blastocyst (0.00% ± 0.00% vs. 49.00% ± 2.65%, *P* < 0.0001) compared to control oocytes (Fig. 3E-G). Collectively, our observations suggest that the embryo development potential is significantly impaired due to the decreased oocyte quality with meiotic defects caused by HAT1 inhibition in GCs.

### HAT1 inhibition causes differential genes expression in oocytes

**Fig. 4:**
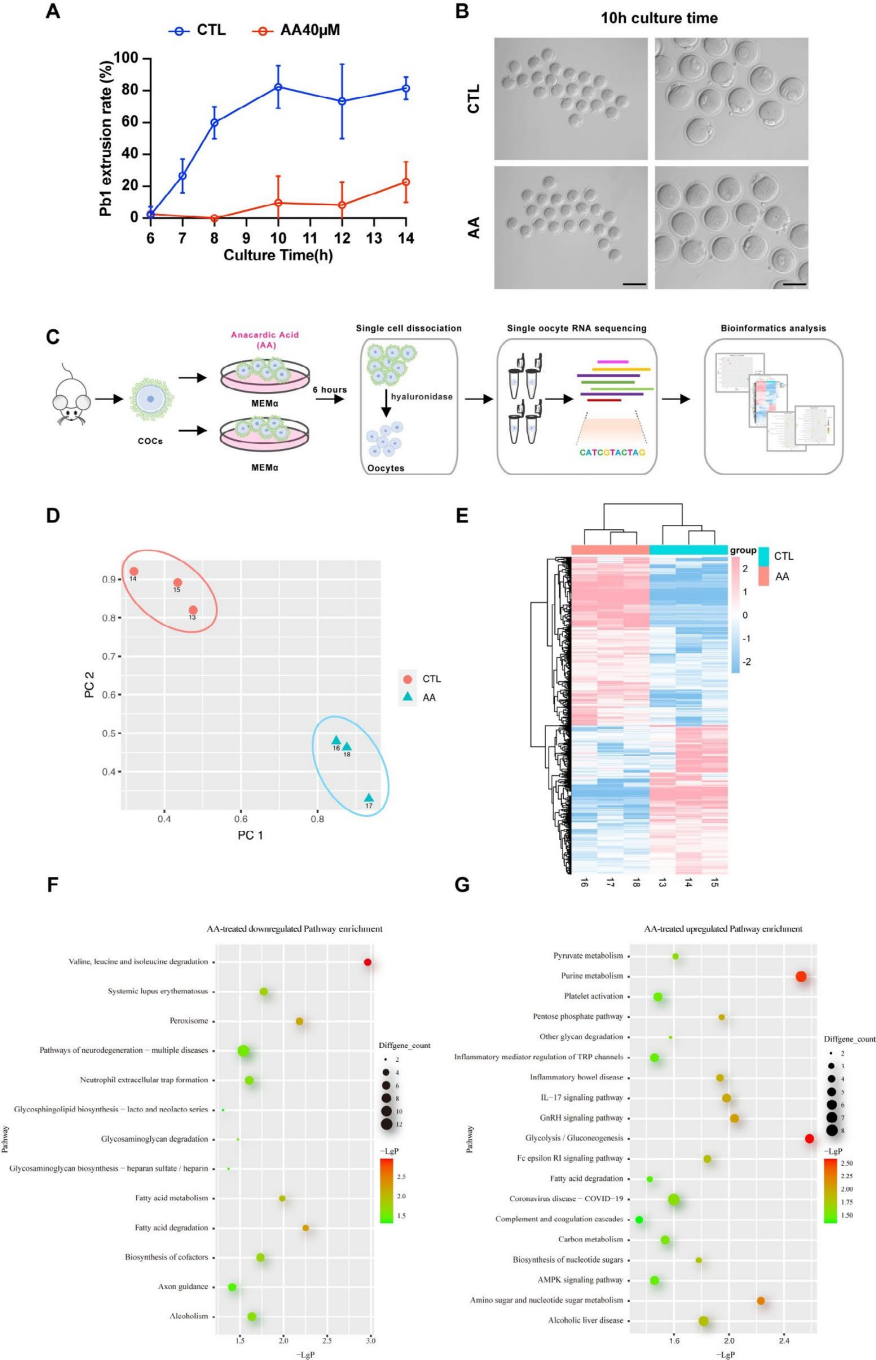
HAT1 inhibition causes differential gene expression in oocytes. **(A)** The rate of PBE at different times from 6 to 14 h in oocytes from the CTL and AA groups. The data are shown with at least three independent experiments at each time point. **(B)** Representative micrographs of the PBE oocytes at 10 h culture time in the CTL and AA groups. Left, Scale bar: 200 μm. Right, Scale bar: 100 μm. **(C)** Flowchart overview of mice single oocyte RNA sequencing in the CTL and AA groups. **(D)** PCA of the transcriptome of RNA sequencing data in oocytes collected from the CTL and AA groups. **(E)** Heatmap showing differential gene expression in the CTL and AA groups. **(F)**-**(G)** Representative GO enrichment analysis of signal pathways of the downregulated pathways (left) and upregulated pathways (right) between the CTL and AA groups

To demonstrate the oocyte gene expression changes after HAT1 inhibition, bioinformatics analysis of oocyte transcriptome sequencing was performed. We first analyzed the time dependence of the PBE in the control and AA-treated groups to select the appropriate sequencing time point. IVM results showed that there was nearly no PB1 extrusion between the two groups at 6 h. After that, oocytes from the control group started to extrude PB1, while AA-treated oocytes failed to extrude PB1 before 10 h (Fig. 4A, B). Therefore, we performed single oocyte RNA sequencing on oocytes from control and AA-treated groups at 6 h of IVM (Fig. 4C).

Principal component analysis (PCA) revealed a dynamic gene expression change that occurred in oocytes of two groups (Fig. 4D). There were 506 upregulated differentially expressed genes (DEGs) and 448 downregulated DEGs comparing the transcriptomes of oocytes from two groups (Fig. 4E). Gene Ontology (GO) analysis revealed that metabolism-related pathways, including “valine, leucine, and isoleucine degradation”, “fatty acid metabolism”, and “fatty acid degradation”, were downregulated in oocytes from AA-treated groups (Fig. 4F). And inflammation-related pathways, including “IL-17 signaling pathway” and “inflammatory mediator regulation of TRP channels” were upregulated (Fig. 4G). These analyses suggest that although GVBD could occur normally in both the control and experimental group at 6 h, there is already differential gene expression, which might be the cause of the aberrant extrusion of the PB1 later.

#### Depletion of HAT1 increases GCs apoptosis and downregulates acetylated FoxO1 expression

**Fig. 5:**
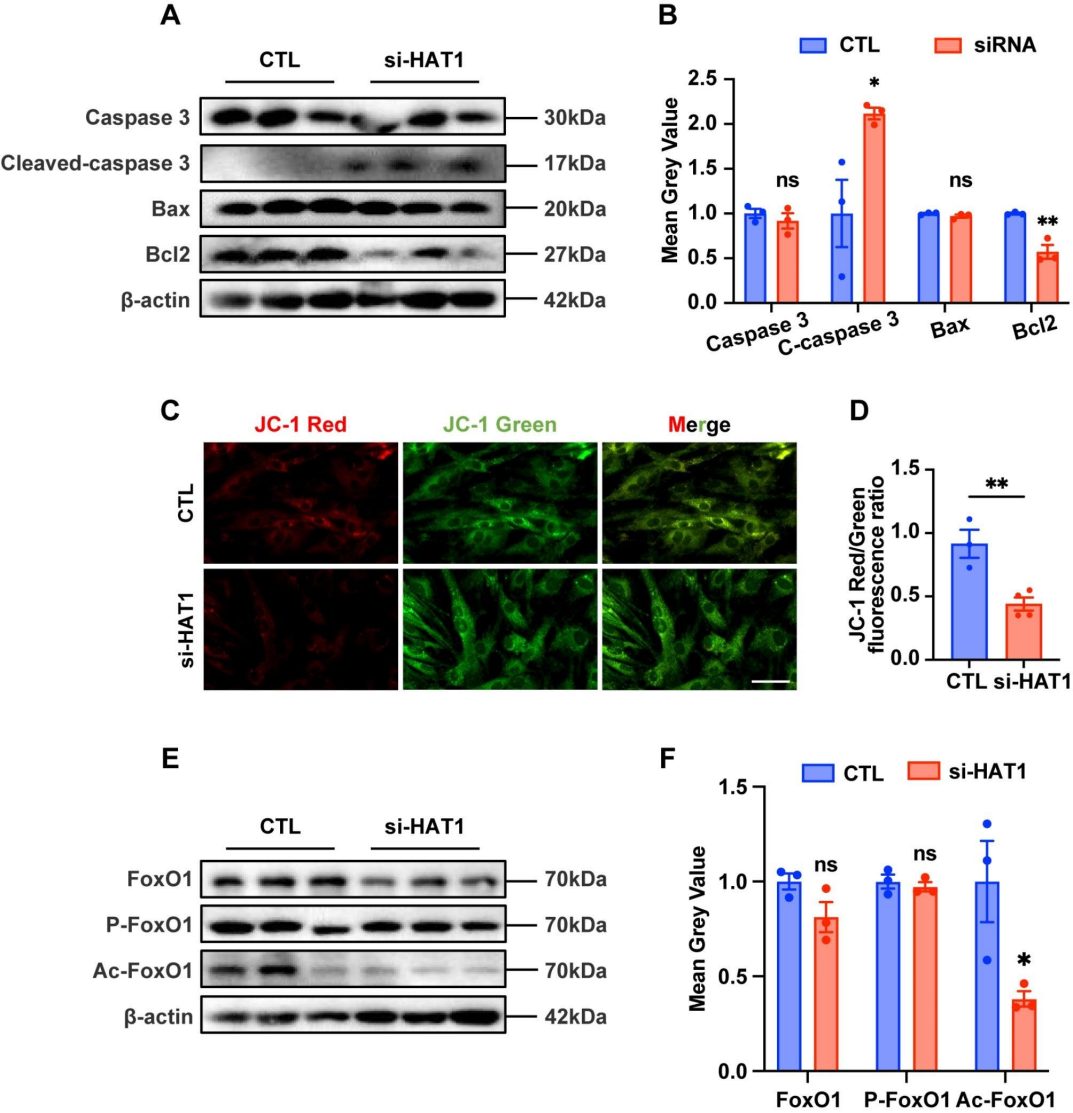
Depletion of HAT1 increases GCs apoptosis and downregulates acetylated FoxO1 expression. **(A)**-**(B)** Western blot analysis of the expression of caspase 3, cleaved-caspase 3, Bax, and Bcl2 in the CTL and si-HAT1 treated KGN cells. The samples used to examine both the efficiency of si-HAT1 knockdown (Fig. 2E) and the level of apoptosis (Fig. 5A) were from the same batch of KGN cells, which leads to the same loading control. The experiments were repeated three times independently with similar results. **(C)** Representative micrographs of the CTL and si-HAT1 treated KGN cells stained with JC-1. Scale bar: 100 μm. **(D)** Ratios of red: green JC-1 fluorescence in the CTL and si-HAT1 groups. The experiments were repeated three times independently with similar results. **(E)**-**(F)** Western blot analysis of the expression of FoxO1, p-FoxO1, and Ac-FoxO1 in the control and si-HAT1 group. The experiments were repeated three times independently with similar results. Data are shown as the mean ± SEM, ns, no significance, *P < 0.05, **P < 0.01, Student’s t test

Since HAT1 inhibition could trigger cell apoptosis and decrease mitochondrial quality [14, 19], we examined cell apoptosis and mitochondrial quality in HAT1 inhibition human GC-like line KGN cells. As shown in Fig. 5A and B, the total level of caspase 3 protein was not changed. However, the knockdown of HAT1 in KGN cells substantially activated cleaved-caspase 3 and suppressed the expression of Bcl2. In addition, as the early feature of programmed cell death, impairment of active mitochondria was detected by JC-1 assay, which reflects the MMP. The results showed that MMP was significantly decreased after HAT1 depletion (Fig. 5C, D). Since FoxO1 is highly expressed in GCs of atretic follicles and plays essential roles in cell apoptosis [20, 21], we assessed the protein expression of total FoxO1, phosphorylated FoxO1, and acetylated FoxO1 in HAT1 knockdown GCs. We found that the expression of acetylated FoxO1 in GCs decreased significantly after HAT1 knockdown, suggesting that HAT1 could acetylate FoxO1 (Fig. 5E, F). The above outcomes illustrate that HAT1 depletion could decrease acetylated FoxO1 and increase GCs apoptosis.

### Depletion of HAT1 decreases FoxO1 nuclear location and downregulates AREG expression in GCs

**Fig. 6:**
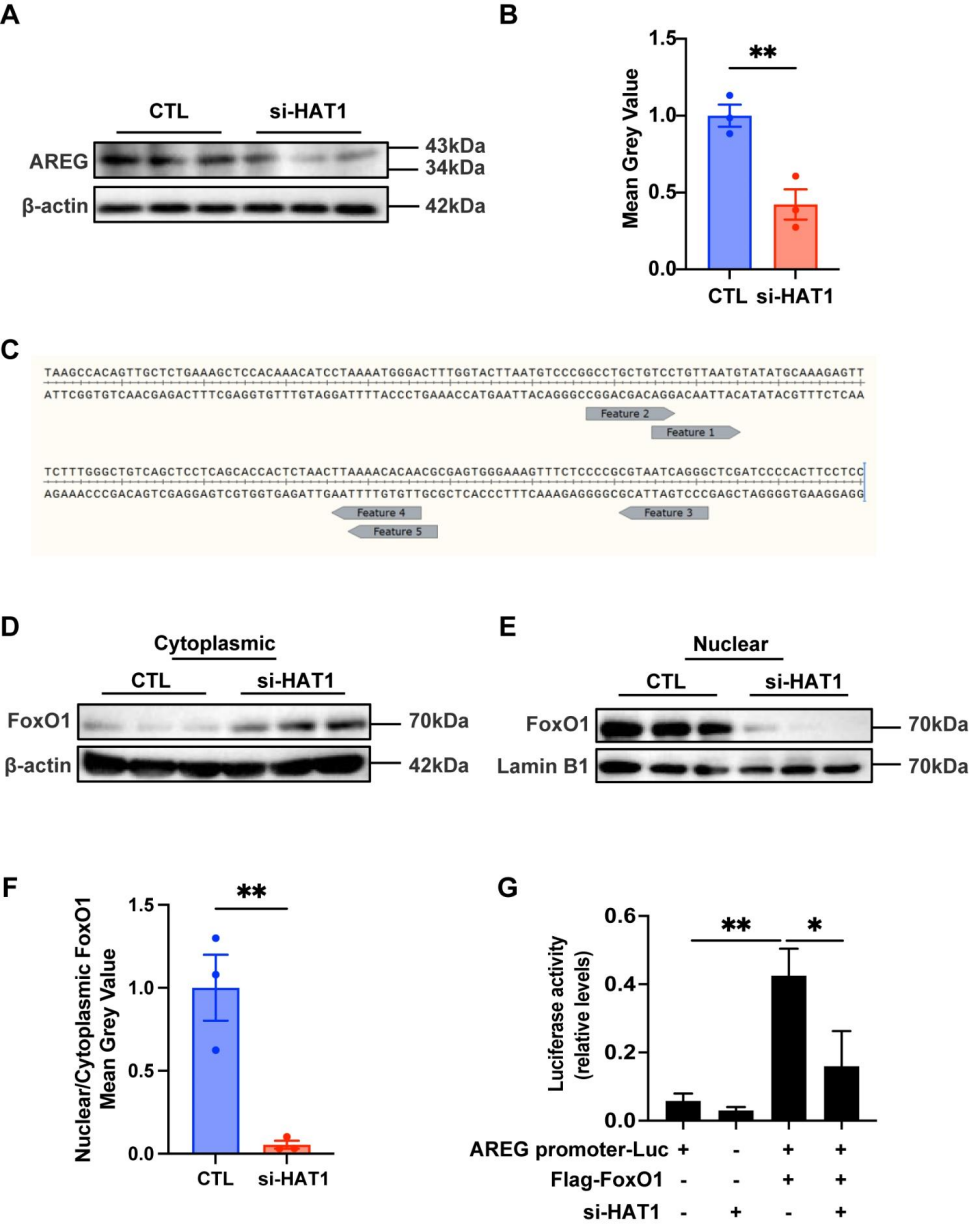
Depletion of HAT1 decreases FoxO1 nuclear location and downregulates AREG expression in GCs. **(A)**-**(B)** Western blot analysis of the expression of AREG in the CTL and si-HAT1 treated KGN cells. The experiments were repeated three times independently with similar results. **(C)** Five possible binding sites of the AREG promoter region and FoxO1. **(D)**-**(F)** Western blot analysis of cytosolic and nuclear fractions in the control and si-HAT1 group. The experiments were repeated three times independently with similar results. **(G)** Dual-luciferase reporter genes assay of AREG promoter and FoxO1. Data are shown as the mean ± SEM, ns, no significance, *P < 0.05, **P < 0.01, Student’s t test

To unravel the mechanisms of downregulation of acetylated FoxO1 in GCs on oocyte meiotic progress, we evaluated the impact of HAT1 knockdown on AREG expression. Since the EGF signal is the most important pathway to regulate oocyte maturation in GCs [22], and AREG, the most abundant EGF in GCs, can induce oocyte meiosis resumption [23]. We found that AREG decreased significantly in HAT1 knockdown GCs (Fig. 6A, B). In addition, we used JASPAR to predict and found that the AREG promoter region contains multiple FoxO1 binding sites (Fig. 6C). We also observed that HAT1 knockdown reduced the nuclear level of FoxO1, suggesting the translocation of FoxO1 into cytoplasmic after HAT1 depletion (Fig. 6D-F). Of note, dual-luciferase reporter genes assays showed that FoxO1 binds to the AREG promoter region to promote AREG expression, while depletion of HAT1 significantly reduced FoxO1-AREG binding (Fig. 6G). The above results indicate that HAT1 acetylates FoxO1 and promotes AREG expression in GCs, which contributes to oocyte maturation.

## Discussion

**Fig. 7:**
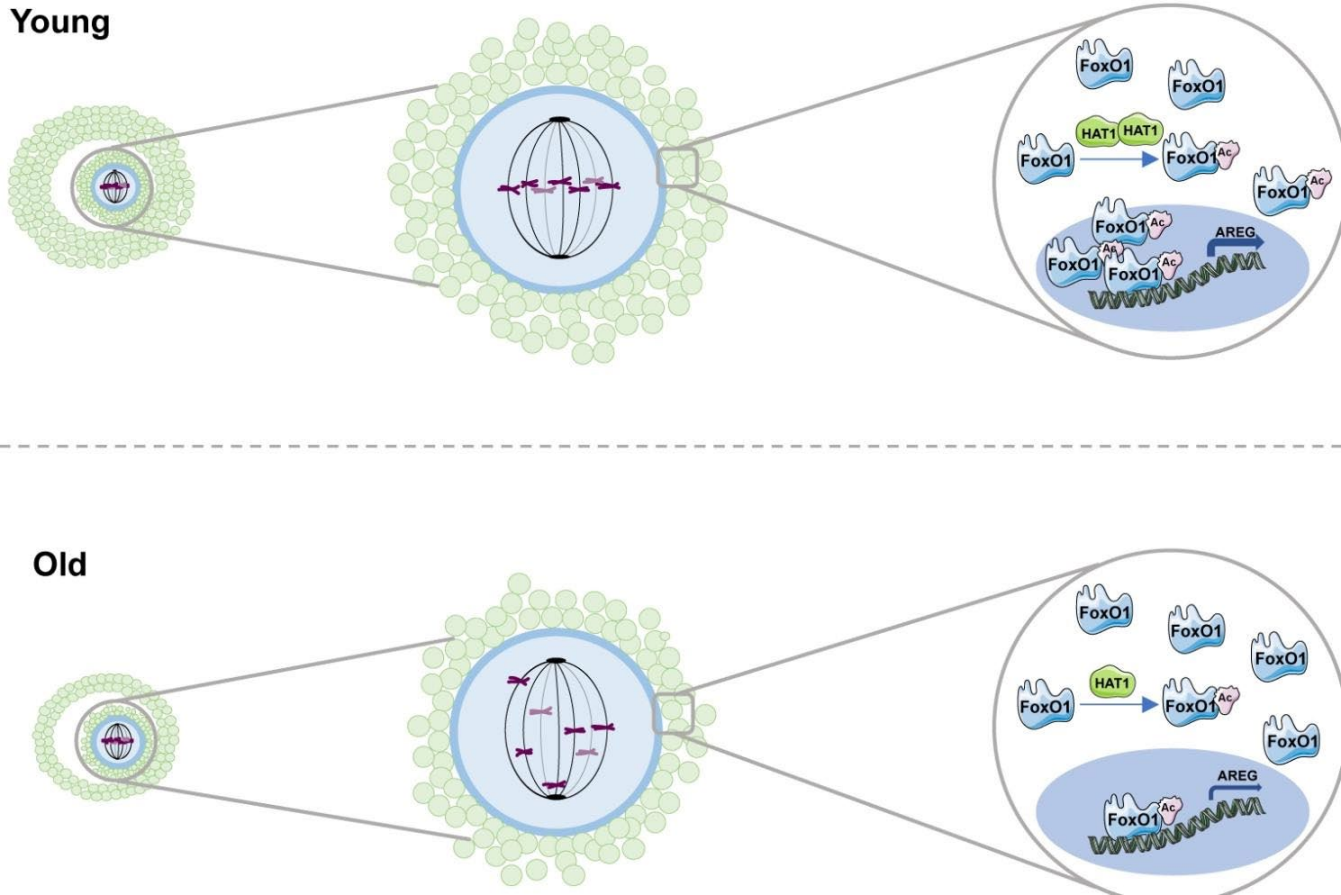
Schematic of the downregulated HAT1 in GCs dominating oocyte meiotic defects and aneuploidy. Under normal circumstances, HAT1 in GCs maintains the normal meiosis process of oocytes by acetylating FoxO1 to regulate AREG expression. HAT1 declines in GCs with maternal age, which causes decreased AREG expression via downregulation of acetylated FoxO1, further disrupting oocyte meiotic progression and causing aneuploidy

Increased aneuploidy due to abnormal oocyte meiosis process is an important reason for decreased oocyte quality in aged women [4, 24, 25]. GCs regulate the progress of oocyte meiosis through signal transduction and metabolic coupling [26]. Abnormal gene expression in GCs leads to oocyte meiotic defects and aneuploidy [9]. In the present study, we found that the expression of HAT1 in GCs decreased with age, and inhibition of HAT1 activity in GCs increased oocyte meiotic defects and aneuploidy. Mechanistic assays demonstrated that HAT1 could acetylate FoxO1, leading to the translocation of FoxO1 into the nucleus and binding to the AREG promoter region, then increasing the expression of AREG (Fig. 7). This study revealed that decreased HAT1 in GCs during ovarian aging is a key regulator of oocyte meiotic defects and aneuploidy.

The acetylation and deacetylation of histone are essential for oocyte maturation. Several studies showed the abnormal expression of acetylation-associated genes in aged ovaries and oocytes and many histone deacetylases (HDACs) members have been reported to regulate oocyte meiosis progress [27–29]. Wang et al. found that HDAC3 in GCs maintains oocyte meiosis arrest by repressing AREG expression, indicating that the acetylation modification in GCs plays an important role in oocyte meiosis resumption [30]. However, the role of acetylation modification remained unclear in GCs during oocyte maturation. According to our study, inhibition of HAT1-mediated acetylation did not affect the meiotic process of DOs but could decrease the meiosis-associated signals in GCs, which disturbed the oocyte maturation and quality.

In this study, we used AA (a histone acetyltransferase that inhibits HAT activity selectively) and siRNAs to test the function of HAT1 during oocyte maturation [31, 32]. However, despite the reduction of PBE and meiotic defects rate, the differences were slight in siRNAs treated COCs compared with the inhibition of HAT1 by AA. We analyze that this might be because oocytes are wrapped with multilayer GCs, which leads to poor transfection efficiency of siRNAs. There is no guarantee that siRNAs can be transfected into all GCs, so the degree of effect of HAT1 knockout on oocyte functional changes is relatively low. Nevertheless, both inhibition and knockout experiments have demonstrated that HAT1 in GCs is crucial for the regulation of the oocyte meiosis process.

HAT1 inhibition could trigger cell apoptosis and decrease mitochondrial quality [14, 16]. Knockdown of HAT1 in KGN cells affected the expression of apoptosis-related genes cleaved-caspase 3 and Bcl2 and decreased MMP. FoxO1 is an important transcription factor that regulates apoptosis in GCs [21]. Studies showed that FoxO1 was highly expressed in GCs of atresia follicles [20]. It has been confirmed that phosphorylation modification of FoxO1 plays an important role in the process of cell apoptosis [33, 34]. Our studies showed that HAT1 knockout increased the GCs apoptosis, but there is no difference in the protein level of FoxO1 and phosphorylated FoxO1. It is well known that the activity of FoxO1 is regulated by both phosphorylation and acetylation, but this regulatory model is controversial. Both acetylation and deacetylation may activate FoxO1 [35–39]. Our study found that the level of acetylated FoxO1 in GCs significantly decreased after the knockdown of HAT1, suggesting that HAT1 can acetylate the non-histone protein FoxO1.

In addition, the EGF signal is the most important pathway to regulate oocyte maturation in GCs [22], and AREG, the most abundant EGF in GCs, can induce oocyte meiosis resumption [23]. Chen et al. found an increased rate of abnormal spindle morphology in MII oocytes of AREG knockdown mice, suggesting that the inactivation of AREG leads to meiosis defects. They also found that AREG depletion affected downstream transcripts including the main functional categories of metabolism, embryonic development, cell cycle, and RNA regulators [40]. The AREG expression in HAT1 knockdown GCs and JASPAR implicated the relationship between HAT1, FoxO1 and AREG. The nucleocytoplasmic separation experiment further demonstrated that FoxO1 in the cytoplasm increased and FoxO1 in the nucleus significantly decreased after the knockdown of HAT1 in GCs, and dual-luciferase reporter genes assay demonstrated the depletion of HAT1 reduced FoxO1-AREG binding. The above results indicate that GCs HAT1 can acetylate and modify the non-histone protein FoxO1 and promote its entry into the nucleus, which is significant for the survival of GCs and AREG expression.

There are some limitations and weaknesses in this study. Firstly, instead of GCs, the KGN cell line was used to study mechanisms for the reason of the difficulty of large numbers of GCs obtaining and survival of many generations. KGN cell line has long-term and stable proliferation [41] and maintains the physiological characteristics of GCs [42]. Moreover, the KGN cell line has been widely used in the study of the function and regulatory mechanisms of GCs biological factors. However, despite the commonly use of KGN cell line for mechanism study, it is not as good as direct study with GCs. Besides, HAT1 has been proven to be closely related to aging. HAT1^+/−^ mice have significantly shortened lifespans and exhibit multiple premature aging phenotypes [14]. We found that the expression of HAT1 was significantly lower in aged mice GCs than in young GCs. The decreased expression of HAT1 in GCs was one reason for abnormal meiosis and the low quality of aged oocytes. This study provides a potential therapeutic target to increase the aged oocyte quality by upregulating the downregulated expression of HAT1 in aged GCs. However, no effective HAT1 agonists have been reported, so this study lacks relevant rescue experiments. New HAT1 agonists await further study.

## Conclusion

In conclusion, we established that HAT1 plays a crucial role in oocyte maturation. The expression of HAT1 in GCs shows a marked decline with maternal age. HAT1 inhibition decreases the AREG expression in GCs and then disturbs the oocyte meiosis progress. Our study indicates a potential target for improving the aged oocyte quality.

### Electronic supplementary material

Below is the link to the electronic supplementary material.

Additional file 1: fig. S1. Immunohistochemistry for HAT1 in ovary slides from 6-week-old mice, 6-week-old mice treated with PMSG 48 h and hCG 4 h, 10-month-old mice, and 10-month-old mice treated with PMSG 48 h and hCG 4 h. Scale bar: 500 μm. The amplified views of the boxed area are shown at the bottom. Scale bar: 100 μm. The experiments were repeated three times independently with similar results.

Additional file 2: fig. S2. **(A)**-**(B)** Quantitative analysis of GVBD and PBE rates in the CTL (n = 45), 10 μm AA-treatment (n = 51), and 20 μm AA-treatment (n = 43) groups. CTL: MEMα maturation medium; 10 µM AA: MEMα maturation medium with 10 µM AA; 20 µM AA: MEMα maturation medium with 20 µM AA. **(C)** Morphology of spindles and chromosomes in oocytes of the CTL (n = 63) and si-HAT1 (n = 56) groups. Scale bar: 25 μm. **(D)** Statistical analysis of meiotic defects rate in the CTL and si-HAT1 groups. The data are shown with five independent experiments. Data are shown as the mean ± SEM, ns, no significance, *P < 0.05, **P < 0.01, Student’s t test.

Supplementary Material 3

## Data Availability

The original data presented in the study are included in the article. Further inquiries can be directed to the corresponding authors.

## Abbreviations

HAT1: Histone acetyltransferase 1
AA: Anacardic Acid
siRNAs: Small interfering RNAs
COCs: Cumulus-oocyte complexes
GVBD: Germinal vesicle breakdown
PBE: The first polar body extrusion
GC: Granulosa cell
GCs: Granulosa cells
FoxO1: Forkhead box transcription factor O1
AREG: Amphiregulin
cGMP: Cyclic guanosine monophosphate
NPPC: Natriuretic peptide precursor type C
NPR2: Natriuretic peptide receptor 2
LH: Luteinizing hormone
LHR: LH receptor
EGF: Epidermal growth factor
EREG: Epiregulin
DOs: Denuded oocytes
SPF: Specific pathogen-free
PMSG: Pregnant mare serum gonadotropin
hCG: Human chorionic gonadotropin
qRT-PCR: Quantitative real-time polymerase chain reaction
RIPA: Radioimmunoprecipitation assay
PVDF: Polyvinylidene difluoride
ECL: Chemiluminescence
IVF: In vitro fertilization
IVM: In vitro maturation
si-HAT1: siRNAs targeting HAT1
DTT: Dithiothreitol
HTF: Human tubal fluid
DAVID: Database for Annotation, Visualization, and Integrated Discovery
MMP: Mitochondrial membrane potential
TF: Transcription factor
FSH: Follicle-stimulation hormone
PCA: Principal component analysis
DEGs: Differentially expressed genes
GO: Gene ontology
HDACs: Histone deacetylases
