# Supplementary figures and images for: Decreased HAT1 expression in granulosa cells disturbs oocyte meiosis during mouse ovarian aging
Source: Reprod Biol Endocrinol. 2023 Oct 31;21:103. doi: 10.1186/s12958-023-01147-w (PMC10617186; doi:10.1186/s12958-023-01147-w)

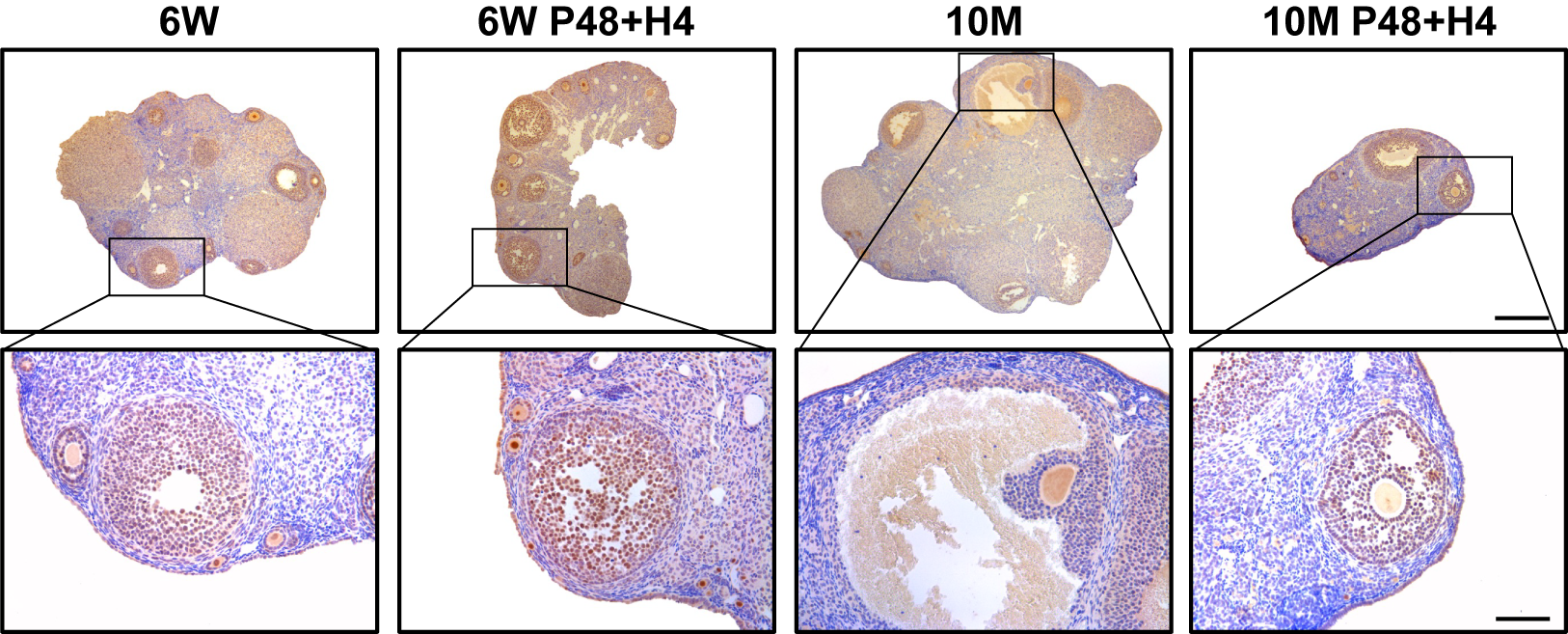

Supplement: Supplementary file 1 — Additional file 1: fig. S1. Immunohistochemistry for HAT1 in ovary slides from 6-week-old mice, 6-week-old mice treated with PMSG 48 h and hCG 4 h, 10-month-old mice, and 10-month-old mice treated with PMSG 48 h and hCG 4 h. Scale bar: 500 μm. The amplified views of the boxed area are shown at the bottom. Scale bar: 100 μm. The experiments were repeated three times independently with similar results. [file 12958_2023_1147_MOESM1_ESM.tif]

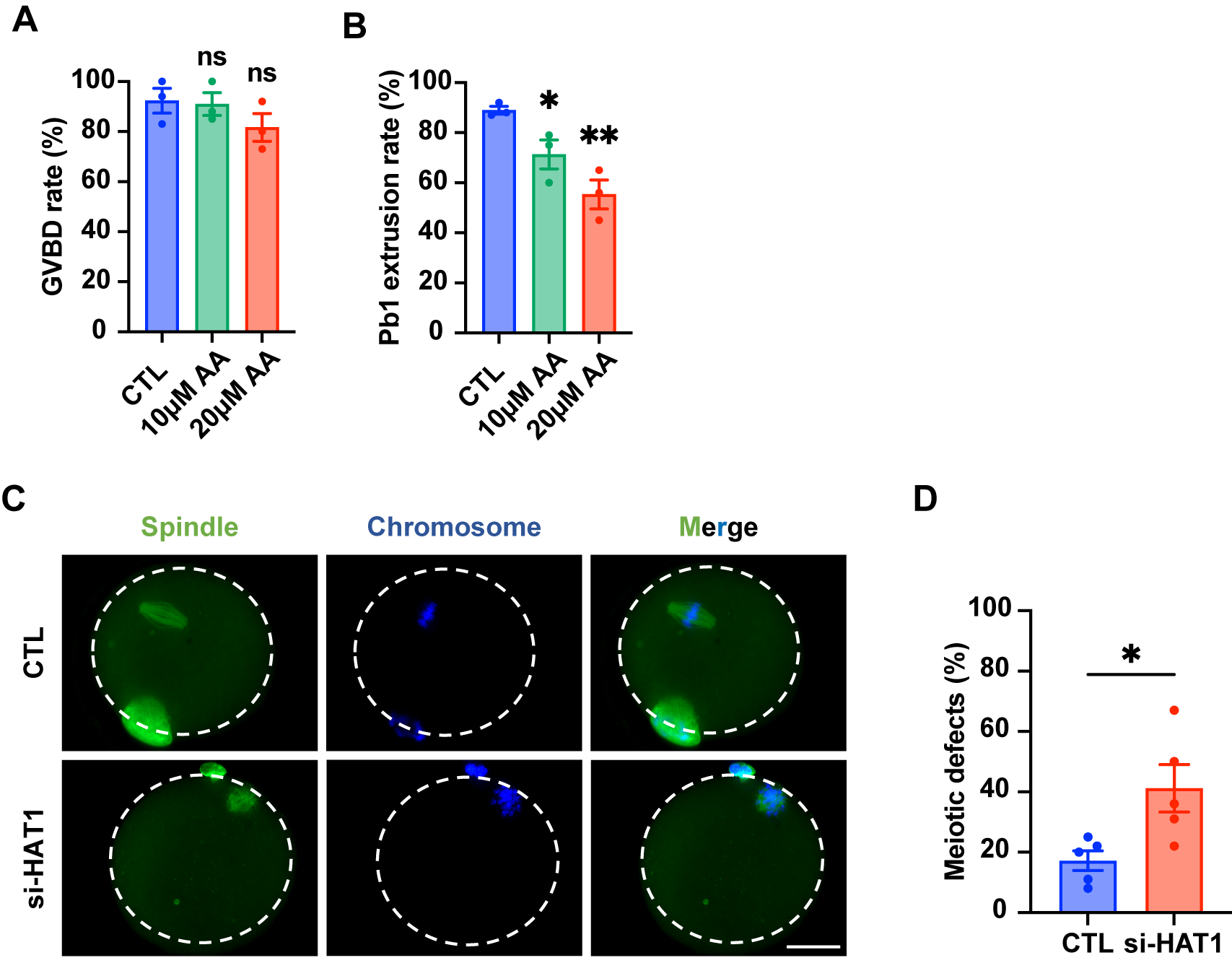

Supplement: Supplementary file 2 — Additional file 2: fig. S2. (A)-(B) Quantitative analysis of GVBD and PBE rates in the CTL (n = 45), 10 μm AA-treatment (n = 51), and 20 μm AA-treatment (n = 43) groups. CTL: MEMα maturation medium; 10 µM AA: MEMα maturation medium with 10 µM AA; 20 µM AA: MEMα maturation medium with 20 µM AA. (C) Morphology of spindles and chromosomes in oocytes of the CTL (n = 63) and si-HAT1 (n = 56) groups. Scale bar: 25 μm. (D) Statistical analysis of meiotic defects rate in the CTL and si-HAT1 groups. The data are shown with five independent experiments. Data are shown as the mean ± SEM, ns, no significance, *P < 0.05, **P < 0.01, Student’s t test. [file 12958_2023_1147_MOESM2_ESM.tif]
