# Supplementary material for: Decreased HAT1 expression in granulosa cells disturbs oocyte meiosis during mouse ovarian aging
Source: Reprod Biol Endocrinol. 2023 Oct 31;21:103. doi: 10.1186/s12958-023-01147-w (PMC10617186; doi:10.1186/s12958-023-01147-w)
Supplement: Supplementary file 3 — Supplementary Material 3 [file 12958_2023_1147_MOESM3_ESM.docx]

**Figure 1B: Western blotting results of HAT1 (left), β-actin (right)**

**
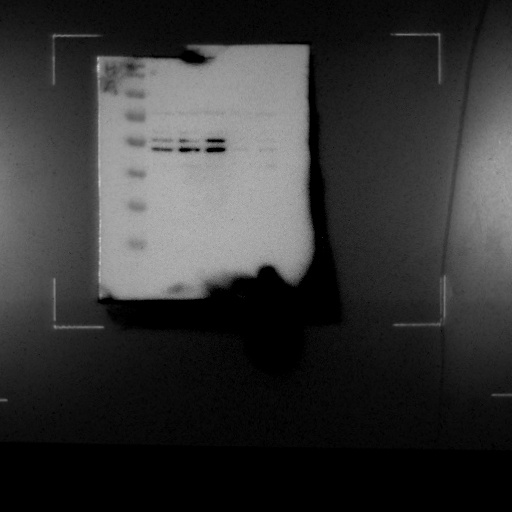

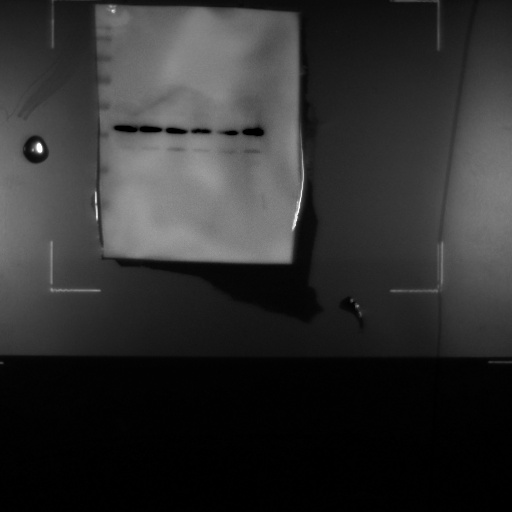
**

**Figure 2E: Western blotting results of HAT1 (left), β-actin (right)**


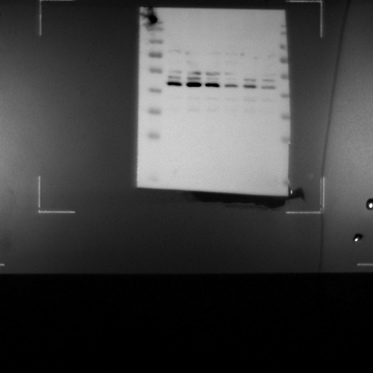

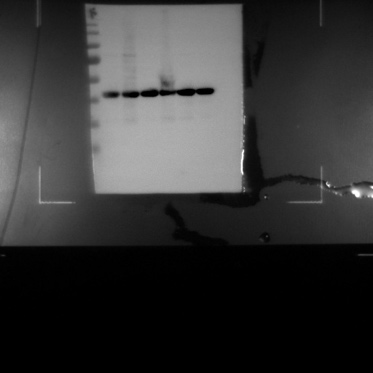


**Figure 5A: Western blotting results of caspase 3 (a), cleaved-caspase 3 (b), Bax (c), Bcl2 (d) and β-actin (e)**

**(a)**
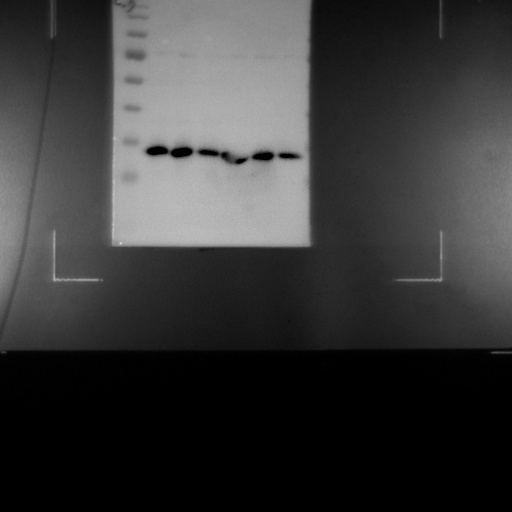
**(b)**
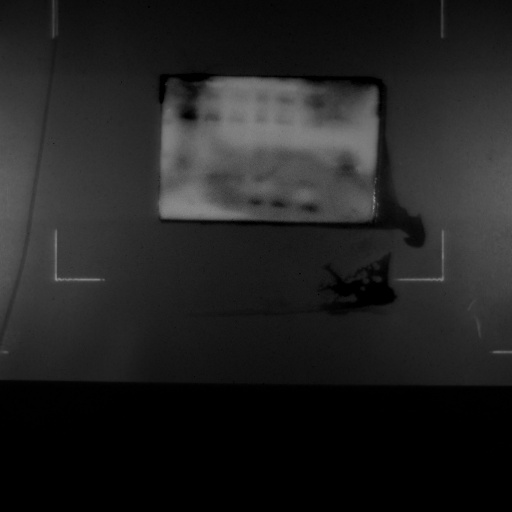
 **(c)**
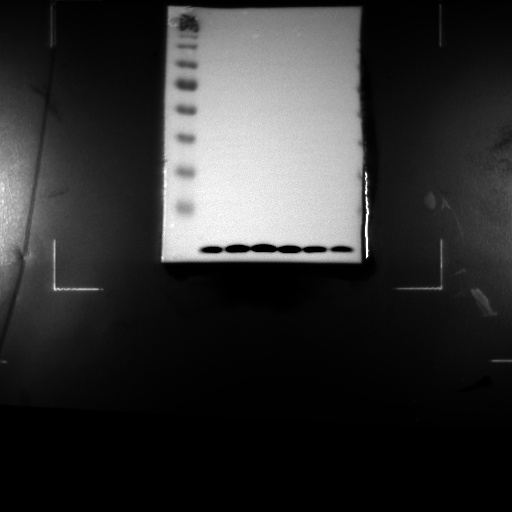
**(d)**
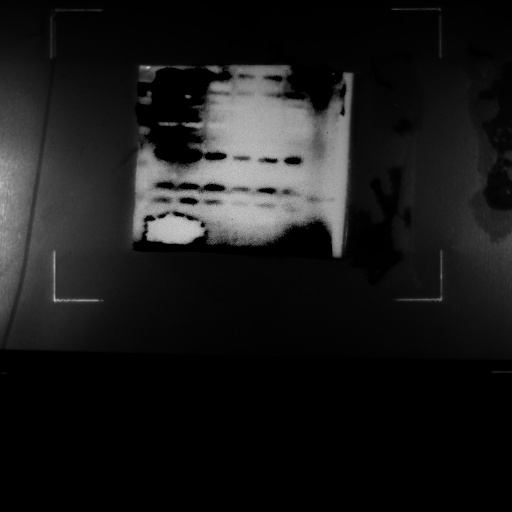
 **(e)**
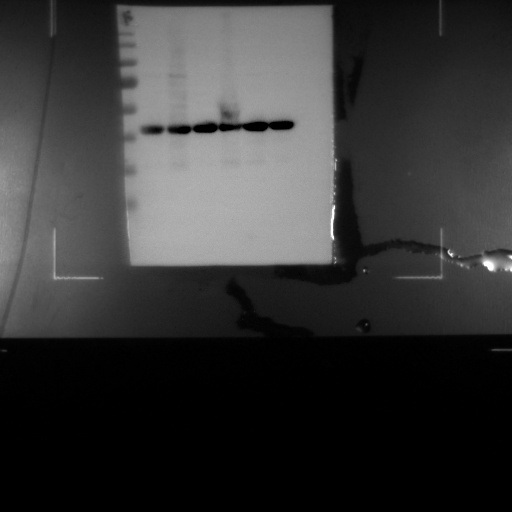


**Figure 5E: Western blotting results of FoxO1 (a), p-FoxO1 (b), Ac-FoxO1 (c) and β-actin (d)**

**(a)
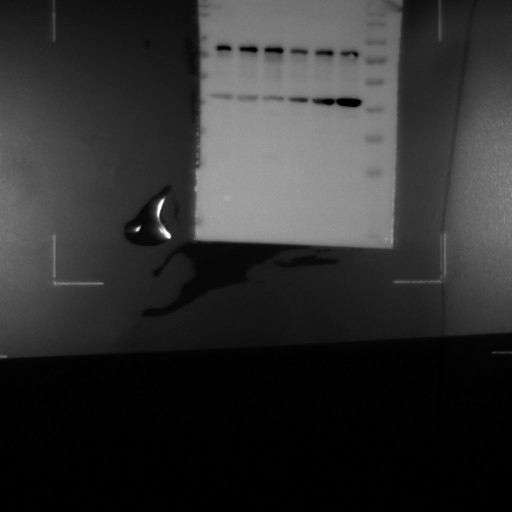
(b)
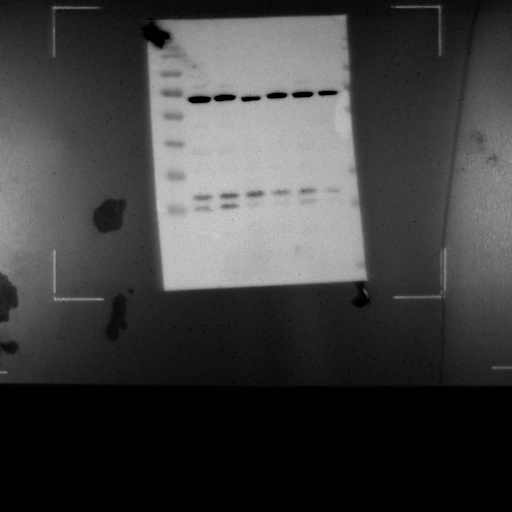
 (c)
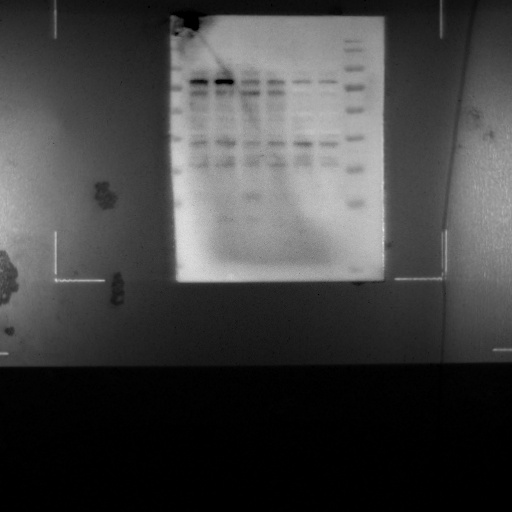
 (d)
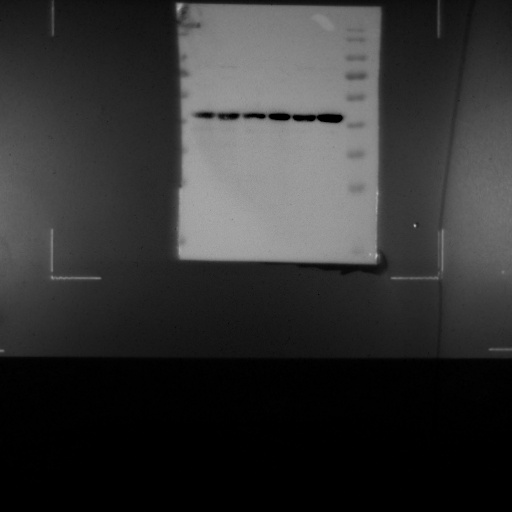
**

**Figure 6A: Western blotting results of AREG (left), β-actin (right)**

**
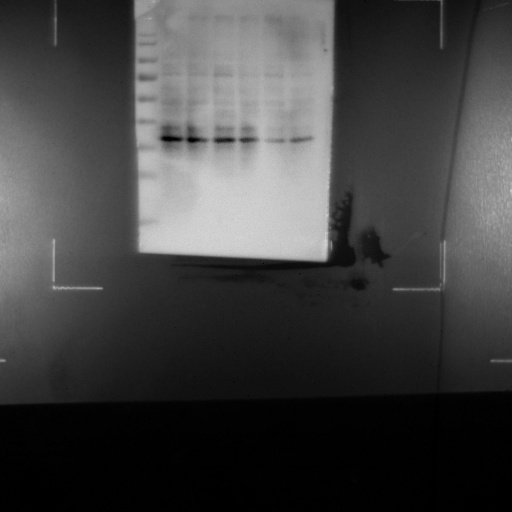

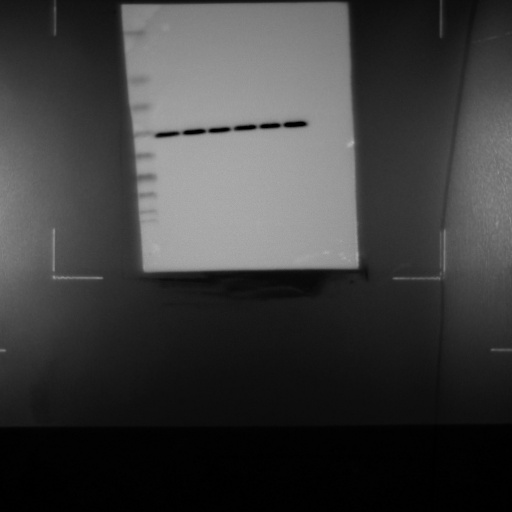
**

**Figure 6D, E: Western blotting results of FoxO1 (a), β-actin (b), and Lamin B1 (c)**

**(a)
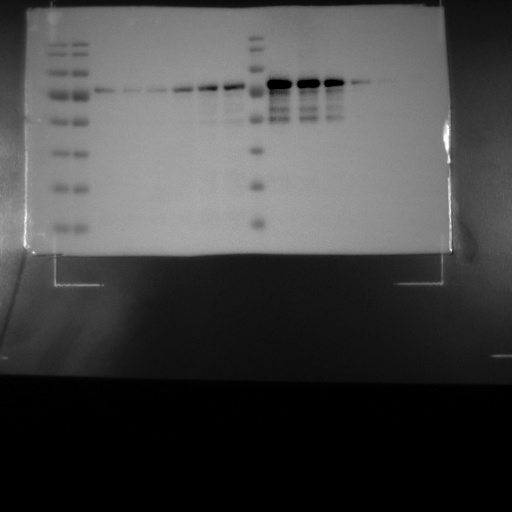
(b)
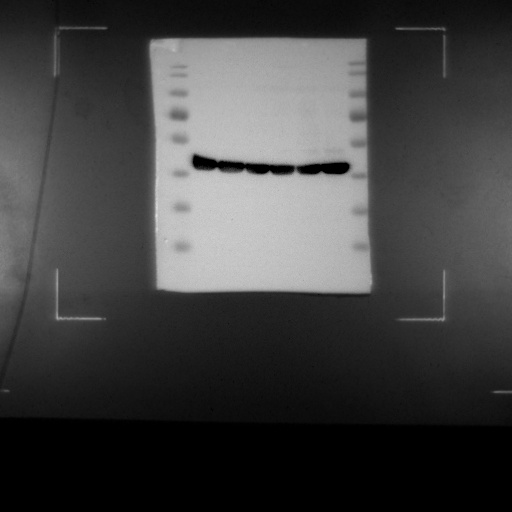
 (c)
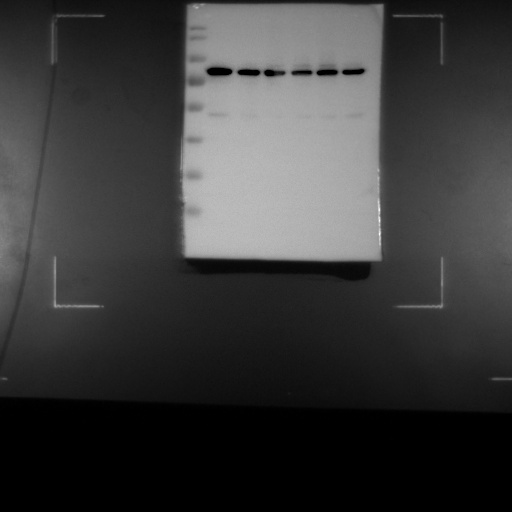
**
